# Detection of epimuscular myofascial forces by Golgi tendon organs

**DOI:** 10.1007/s00221-021-06242-1

**Published:** 2021-10-22

**Authors:** Huub Maas, Wendy Noort, Hiltsje A. Smilde, Jacob A. Vincent, Paul Nardelli, Timothy C. Cope

**Affiliations:** 1 Department of Human Movement Sciences, Faculty of Behavioural and Movement Sciences, Amsterdam Movement Sciences, Vrije Universiteit Amsterdam, Amsterdam, The Netherlands; 2 Department of Neuroscience, Cell Biology and Physiology, Wright State University, Dayton, OH 45435 USA; 3 School of Biological Sciences and Department of Biomedical Engineering, Georgia Institute of Technology, Atlanta, GA USA; 4 The Coulter Department of Biomedical Engineering, Emory University and Georgia Institute of Technology, Atlanta, GA USA

**Keywords:** Golgi tendon organ, Primary afferent, Myofascial force transmission, Rat, Proprioception

## Abstract

Skeletal muscles embed multiple tendon organs, both at the proximal and distal ends of muscle fibers. One of the functions of such spatial distribution may be to provide locally unique force feedback, which may become more important when stresses are distributed non-uniformly within the muscle. Forces exerted by connections between adjacent muscles (i.e. epimuscular myofascial forces) may cause such local differences in force. The aim of this exploratory study was to investigate the effects of mechanical interactions between adjacent muscles on sensory encoding by tendon organs. Action potentials from single afferents were recorded intra-axonally in response to ramp-hold release (RHR) stretches of a passive agonistic muscle at different lengths or relative positions of its passive synergist. The tendons of gastrocnemius (GAS), plantaris (PL) and soleus (SO) muscles were cut from the skeleton for attachment to servomotors. Connective tissues among these muscles were kept intact. Lengthening GAS + PL decreased the force threshold of SO tendon organs (*p* = 0.035). The force threshold of lateral gastrocnemius (LG) tendon organs was not affected by SO length (*p* = 0.371). Also displacing LG + PL, kept at a constant muscle–tendon unit length, from a proximal to a more distal position resulted in a decrease in force threshold of LG tendon organs (*p* = 0.007). These results indicate that tendon organ firing is affected by changes in length and/or relative position of adjacent synergistic muscles. We conclude that tendon organs can provide the central nervous system with information about local stresses caused by epimuscular myofascial forces.

## Introduction

Feedback from Golgi tendon organs plays an important role in the neural control of movement (Akay et al. 2014; Donelan et al. 2009; Gregor et al. 2006; Maas et al. 2007; Rossignol et al. 2006), the regulation of limb stiffness (Nichols 2018) and the senses of force and heaviness (Proske and Gandevia 2012). Tendon organs are located at the junction between a group of skeletal muscle fibers and aponeurosis or tendon (Huber and DeWitt 1900; Zelena and Soukup 1983). When a force is exerted, in response to excitation of the in-series muscle fibers or whole muscle lengthening, the tendon organ will be stretched. The dispersed endings of the afferent (group Ib) axon will be compressed and, consequently, fire (Jami 1992). Therefore, tendon organs are generally known as receptors signaling muscle force. As tendon organs respond predominantly to contractions of muscle fibers that are in the same muscle region (Cameron et al. 1981), they can be considered “regional tension sensors” (Proske and Gandevia 2012).

Skeletal muscles do not have only one tendon organ but many (Jami 1992). The number of tendon organs varies between species and muscles. For instance, cat soleus muscle (SO) has ≈ 45 and rat SO has ≈ 16, while cat peroneus longus has ≈ 16 and rat extensor digitorum longus has ≈ 13 tendon organs (Jami 1992; Zelena and Soukup 1983). Tendon organs are located either at the proximal or distal fiber ends and spatially distributed along the whole length of the aponeuroses (Swett and Eldred 1960; Wohlfart and Henriksson 1960; Zelena and Soukup 1983). Why each muscle needs so many ‘force sensors’ and why tendon organs are located at both the origin and insertion of muscle fibers are unresolved questions.

An answer to the second question might be found in the structural organization of the muscular system. Each anatomically defined skeletal muscle is typically considered as a separate, mechanically independent entity that is connected to the skeleton exclusively at its origin and insertion. Such a system, in which force exerted at the origin or insertion is representative of whole muscle force, arguably implies that a limited number of tendon organs at one muscle end (thus either its origin or insertion) would be sufficient. In the past 20 years, it has become clear that muscles are mechanically not independent, but linked to each other and to non-muscular surrounding structures by connective tissues that are capable of transmitting muscle fiber force (Huijing 2007; Maas 2009; Maas and Finni 2018; Maas and Sandercock 2010). One of the most evident features of such so-called epimuscular myofascial pathways is a difference in force exerted at the origin (proximally) and insertion (distally) of a muscle (Huijing and Baan 2001). Another feature is that length changes in one muscle can affect forces exerted at the tendons of muscles that are kept at a constant length (Bernabei et al. 2015; Maas et al. 2001; Olesen et al. 2014). Due to their proximal–distal distribution, differential feedback from these sensors is expected and, thus, proximal–distal force differences may be sensed by the central nervous system. Such differential information may also be used for joint-specific force feedback from muscles crossing two joints (Maas 2009). At present, there is no evidence of such anatomical organization in the spinal circuitry, such as for intermuscular force feedback (Nichols 2018). A necessary first step in testing this hypothesis is to assess the extent of feedback modulation from tendon organs in response to epimuscular myofascial forces. We recently showed that lengthening a synergistic muscle, and hence the force exerted via epimuscular connections, affected the firing behavior of muscle spindles located within a neighboring muscle (Smilde et al. 2016). Also contraction of synergistic muscles has been reported to unload muscle spindles, and reduce their firing rate, in the agonist muscle (Burke et al. 1976).

The aim of this exploratory study was to investigate the effects of mechanical interactions between adjacent muscles on sensory encoding by tendon organs. For this purpose, firing from single tendon organ afferents in an agonistic muscle were measured intra-axonally for different conditions of synergistic muscles. We hypothesized that within an intact muscle compartment, tendon organ firing is affected by changes in length and/or relative position of adjacent synergistic muscles. First, the same experimental conditions were tested as those of our previous study on muscle spindles (Smilde et al. 2016). Because those conditions involve unphysiological muscle lengths and relative positions, we added a second experiment in which physiological changes in muscle length and relative position of ankle plantar-flexors were imposed by simulating ankle joint and/or knee joint rotations.

## Methods

### Overview of experimental design

This study consists of two experiments in which the length and relative position of ankle plantar-flexors were manipulated. In the first experiment (Experiment 1), firing behavior of tendon organs was assessed in response to stretches of either gastrocnemius and plantaris (GAS + PL) or soleus (SO) muscles distally. In addition, the length of the synergist(s) was manipulated. Note that during normal movements of the ankle joint, these muscles will be lengthened simultaneously and with similar magnitudes. Therefore, we added a second experiment (Experiment 2) in which physiological changes in muscle length and relative position were imposed by exploiting the anatomy of the ankle plantar-flexion muscle group (Maas and Sandercock 2008). GAS and PL are bi-articular muscles crossing both the ankle and knee joint, while SO is a mono-articular muscle crossing only the ankle joint. By lengthening lateral gastrocnemius (LG) and PL, changes in knee joint angle were simulated and the position of LG + PL relative to SO was manipulated in a physiological manner (for details see below).

### Animals

Data were collected from 7 female Wistar rats (body mass = 244 ± 10 g), for Experiment 1 and 8 female Wistar rats (body mass = 250 ± 12.9 g) for Experiment 2. Experiment 1 was performed at Wright State University and approved by its Institute Animal Care and Use Committee (Permit Number: AUP 921). Experiment 2 was performed at the Vrije Universiteit Amsterdam and was approved by the Committee on Ethics of Animal Experimentation at the Vrije Universiteit Amsterdam (Permit Number: FBW 12-02).

For Experiment 1, anesthesia was maintained with isoflurane (1–3% with 100% O2) via a tracheal cannula, such that withdrawal reflexes were fully suppressed. For Experiment 2, rats were anesthetized by an intraperitoneal injection of urethane (initial dose: 1.2 ml/100 g body mass, 12.5% urethane solution; additional doses to maintain suppression of withdrawal reflexes: 0.2 ml). Animal core body temperature (35–37 °C) was maintained with heating pads and lamps. Subcutaneous injections of lactated Ringer solution (1 ml/hour) were given to maintain fluid balance. At the termination of the experiment, rats were euthanized either with an overdose of isoflurane and then exsanguinated via surgical removal of the heart (Experiment 1) or with an overdose of intracardially injected pentobarbital sodium followed by a double-sided pneumothorax (Experiment 2).

### Surgical procedures

**Fig. 1:**
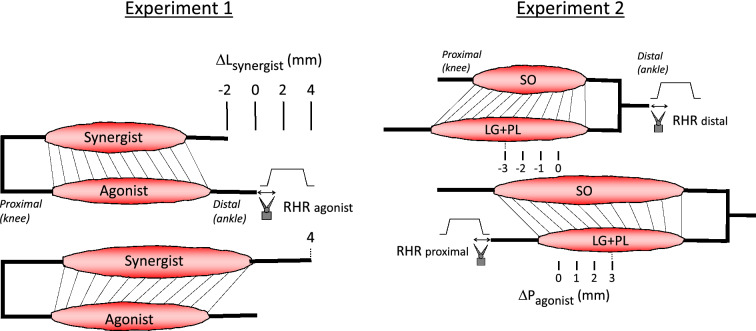
Overview of muscle lengths and relative positions for different experimental protocols. Experiment 1 (left). Diagram of muscle–tendon unit lengths of synergistic muscle(s) when agonist ramp-hold-releases (RHRs) were applied. Soleus muscle (SO) and the combination of gastrocnemius and plantaris muscles (GAS + PL) were alternately agonist and synergist. ΔLsynergist = 0 mm refers to the reference length and position, corresponding to a knee and ankle angle of 90°. Synergist length was changed from Lref – 2 mm to Lref + 4 mm in increments of 2 mm. Connective tissue linkages between the muscles are schematically illustrated as straight lines and shown for synergist length at Lref – 2 mm (top) and synergist at Lref + 4 mm (bottom). Note that only the distal tendons of the muscles were dissected and connected to servomotors (see Surgical procedures). Experiment 2 (right). Diagram of the position of the agonist (LG + PL) relative to the synergist (SO) when agonist RHRs were applied. ΔPagonist = 0 refers to the reference length and position, corresponding to a knee and ankle angle of 90°. For the most proximal relative positions (Pref – 3 mm to Pref), RHRs were applied distally. For the most distal relative positions (Pref to Pref + 3 mm), RHRs were applied proximally. Connective tissue linkages between the muscles are schematically illustrated as straight lines and shown for agonist position Pref – 3 mm (top) and Pref + 3 mm (bottom). Note that the distal tendons of the LG + PL (agonist) and SO (synergist) were tied and connected to one servomotor, and that proximally only the tendons of LG + PL were connected to a servomotor (see “Surgical procedures”).

The surgery for Experiment 1 was equal to that described in a previous study (Smilde et al. 2016) and similar to that of Experiment 2 (the differences are illustrated in Fig. 1). In brief, the lateral and medial gastrocnemius (LG and MG, respectively), SO and PL muscles were dissected free from their surroundings. The biceps femoris muscle and crural fascia were resected, but care was taken to ensure the connective tissues between GAS, SO and PL muscle bellies were left intact. With the knee and ankle angles at 90˚, sutures were placed on the proximal and distal tendons of LG, as well as on the proximal tendon of extensor digitorum longus muscle for the proximal reference position and on the peroneus longus distal tendon for the distal reference position. The corresponding length and relative position will be referred to as reference length (Lref) and reference position (Pref), respectively.

For Experiment 1, the tendons of GAS and SO were separated and a suture was tied to the distal tendons of SO and GAS + PL, respectively. Then the calcaneal bone was cut from the skeleton with the GAS attached to it, and the PL tendon was cut at the level of the calcaneal bone. The distal tendons of GAS + PL and SO were attached to separate servomotors (Aurora Scientific 309C and 305L-BR, respectively) to manipulate and record muscle–tendon unit (MTU) length and measure force at the distal tendons.

For Experiment 2, the medial gastrocnemius muscle (MG) was dissected free. The tendons of LG, PL and SO were tied. The calcaneal bone was cut with LG and SO attached to it, and the PL tendon was cut at the level of the calcaneal bone. Also the proximal tendons of LG and PL were tied and cut. The distal tendons of LG + PL + SO and the proximal tendons LG + PL were attached to separate servomotors (each to Aurora Scientific 309C).

The hindlimb was secured to the set-up with a knee and ankle angle of 90˚. The common peroneal, sural, plantar and MG nerves were isolated and crushed. A bipolar stimulating electrode was placed on the nerve branches of LG and SO. To prevent tissue dehydration, the muscles were irrigated regularly with saline. Prior to back dissection, a local anesthetic (bupivacaine; 1 ml, 0.025%) was injected subcutaneously. An incision was made in the skin (from sacral vertebrae S2 to thoracic vertebrae Th10), and tissues surrounding the spinous and articular processes were removed, so that the spine could be securely clamped. Skin flaps were used to construct a bath of mineral oil. Dorsal roots (L1–L5) were exposed by laminectomy and removal of dura mater, but not cut from the spinal cord.

### Experimental protocols

Stretches in the form of a ramp-hold-release (RHR; 20 mm/s ramp and release, amplitude 3 mm, 1 s hold phase) were applied to the ankle plantar-flexion muscles, which were kept in a passive state. This mimics the muscle conditions during the swing phase of rat locomotion (Bernabei et al. 2017). For all conditions, three consecutive RHRs were applied with 4 s rest in between. Lengthening the MTU of SO, GAS and PL by 3 mm corresponds approximately to a 45˚ change in ankle angle (Johnson et al. 2008). Due to the deep anesthetic plane (see above), no reflex-mediated muscle activity in response to muscle stretch was observed. Ramps were applied at reference length (knee and ankle angle 90˚), or in the absence of a response, at a length at which firing during the stretch and hold phase was found (maximally Lref + 3 mm).

For Experiment 1 (Fig. 1, left), the starting length was Lref + 1.00 ± 0.93 mm for SO and Lref + 0.63 ± 0.74 mm for GAS + PL (mean ± SD). Agonist RHRs were applied distally at four different lengths of the synergistic muscle(s), as obtained by displacements of its distal tendon from Lref – 2 mm to Lref + 4 mm in increments of 2 mm.

For Experiment 2 (Fig. 1, right), the starting length was Lref + 1.36 ± 0.67 mm for LG + PL + SO distal and Lref + 0.82 ± 0.60 mm for LG + PL proximal. RHRs were applied at different relative positions of LG + PL, keeping the MTU length of LG + PL constant. The position of LG + PL was changed from proximal (Pref – 3 mm) to distal (Pref + 3 mm) in steps of 1 mm, as obtained by displacements of the distal and proximal tendons in a distal direction. It should be noted that displacements of the distal tendons also caused changes in MTU length of SO. As our study was aimed at investigating the effects of mechanical interactions between adjacent muscles, and not the effects of MTU length, only LG tendon organs were studied in Experiment 2. For the most proximal relative positions (Pref – 3 mm to Pref), RHRs were applied at the distal tendon. For the most distal relative positions (Pref to Pref + 3 mm), RHRs were applied at the proximal tendon.

### Afferent recordings

The dorsal root containing tendon organs from SO and LG was selected using bipolar recording electrodes. Action potentials were recorded intra-axonally in the dorsal root with a glass micropipette (25–30 MΩ, 2 M K-acetate). Afferents were identified when an orthodromic action potential, in response to an electrical twitch stimulus to the SO and LG nerves, was recorded with a conduction delay of < 3 ms (see Table 1 and 2). Axons were classified as tendon organs if they fired during the force rising phase of an electrically evoked isometric twitch contraction.

**Table 1:** Tendon organ firing and tendon force parameters in Experiment 1 measured before and during ramp-hold-releases of the agonistic muscle with the synergistic muscle at reference length (Lref)

Parameters at Lref	Soleus (*n* = 8)	Lateral gastrocnemius (*n* = 8)
Proximal–distal position	2d, 1d/m, 0 m, 0 pm, 4p, 1nc	1d, 2d/m, 1 m, 2p/m, 0p, 2nc
Axonal conductional delays (ms)	1.6 ± 0.1	1.8 ± 0.2
Force threshold (N)	0.36 ± 0.28	0.76 ± 0.70
Length threshold (mm)	0.99 ± 0.83	1.21 ± 0.73
Peak frequency (pps)	99 ± 48	120 ± 36
Static index (pps)	49 ± 18	53 ± 10
Pre-ramp force agonist (N)	0.10 ± 0.07	0.07 ± 0.03
Max ramp force agonist (N)	0.99 ± 0.56	2.12 ± 0.71

*n* number of afferents, positions: *d* distal, *m* middle, *p* proximal, *nc* not classified

Values are means ± SD

To assess in which muscle (SO or LG) and where in the muscle (proximal, middle or distal) the spindle was located, the muscle surface was probed (Banks et al. 1997). The location that was most sensitive to a small deformation applied with a small tipped glass probe was selected. If probing was not unequivocal, the muscle surface was stimulated (twitches just beyond stimulus threshold) using a bipolar ball electrode.

**Fig. 2:**
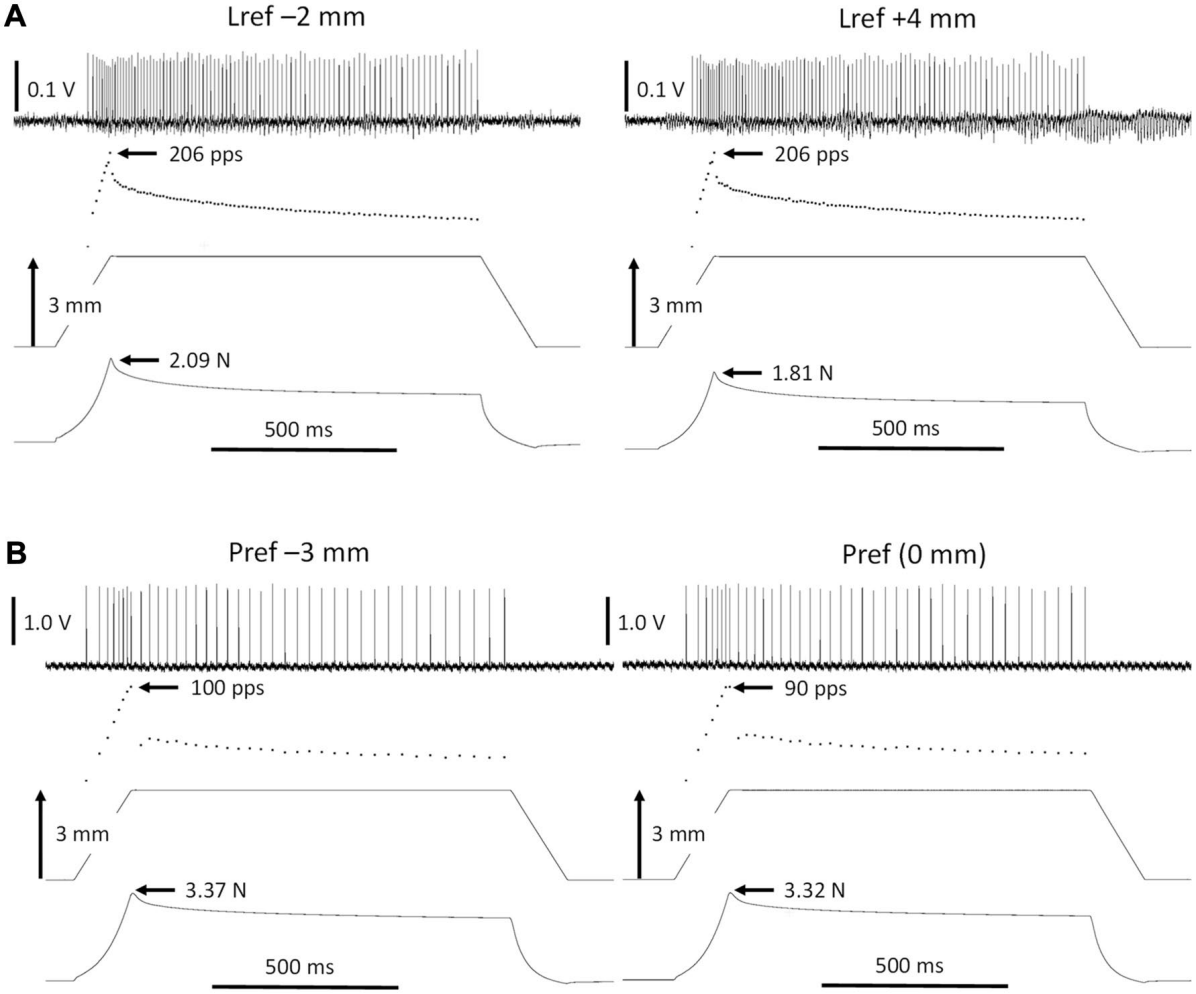
Exemplar recordings for conditions in Experiment 1 (**A**) and Experiment 2 (**B**). In all graphs, the signals from top to bottom are: action potentials, instantaneous firing rate, change in length of agonist, force of agonist. **A** Responses of a soleus (SO) tendon organ during ramp-hold-releases of SO for two lengths of gastrocnemius and plantaris muscles (GAS + PL), i.e. at Lref – 2 mm (left) and at Lref + 4 mm (right). **B** Responses of a lateral gastrocnemius (LG) tendon organ during ramp-hold-releases applied distally to LG + PL and SO for two positions of LG + PL, i.e. Pref – 3 mm (left) and Pref (right). See Fig. 1 for illustrations of the muscle lengths and relative positions for the two experiments

To quantify afferent firing behavior in response to the RHRs, the following parameters were assessed: The maximum instantaneous firing rate (IFR) at the end of the dynamic stretch (peak frequency, PF), the IFR halfway through the hold phase (static index, SI), the force and length thresholds (the force and MTU length change at which the first action potential occurred). The first two parameters were assessed for comparison with the literature. The latter two were used for answering our research question (For exemplar traces see Fig. 2).

Intra-axonal records were amplified (100x) and low-pass filtered (second order, 2 Hz–10 kHz; Axoprobe 1A, Axon Instruments Inc., Synnyvale, CA). All signals were digitized (20 kHz; Power 1401, CED Limited, Cambridge, England) and stored on a computer for later analysis using Spike2 software (CED). Because the firing of tendon organs in response to the first stretch differed from the subsequent stretches, only the latter two of the three RHRs were considered for analysis. For each of the above-described parameters, the mean of those two RHRs was calculated.

Data were excluded if the afferent recording was not stable enough to measure all experimental conditions, when noise prevented reliable discrimination of action potentials, or when control measurements showed history effects. For Experiment 1, afferent responses were recorded from 8 tendon organs located within SO and 8 tendon organs located within LG. For Experiment 2, afferent responses were recorded from 11 tendon organs located within LG.

### Statistics

Data were analyzed using SPSS version 26 (SPSS Inc., Chicago, IL). Repeated measures ANOVA’s were performed; factor length synergistic muscle for Experiment 1, factor relative position of agonistic muscle for Experiment 2. Because RHRs were applied at different tendon ends for the most proximal (Pref – 3 mm to Pref) and the most distal relative positions (Pref to Pref + 3 mm), the corresponding results for the proximal muscle positions were analyzed separately from the results for the distal positions. If the assumption of sphericity was violated, assessed using Mauchly's sphericity test, Greenhous–Geisser correction was applied. The statistically significant level was set at *p* = 0.05. If a significant group effect was found, post-hoc analysis with a Bonferonni correction was performed.

## Results

### Experiment 1: effects of distal length changes of synergistic muscles

#### Characteristics of recorded tendon organ afferents

Axonal conductional delays and ramp parameters, measured with the synergist muscle at Lref (Table 1), were consistent with those described previously (Bullinger et al. 2011; Vincent et al. 2017). The approximate location within the muscle belly could be identified for all except 1 out of 8 SO afferents and 2 out of 8 LG afferents, but all afferents were included in the analysis.

#### Force changes in agonistic muscle in response to length changes of synergistic muscle(s)

**Fig. 3:**
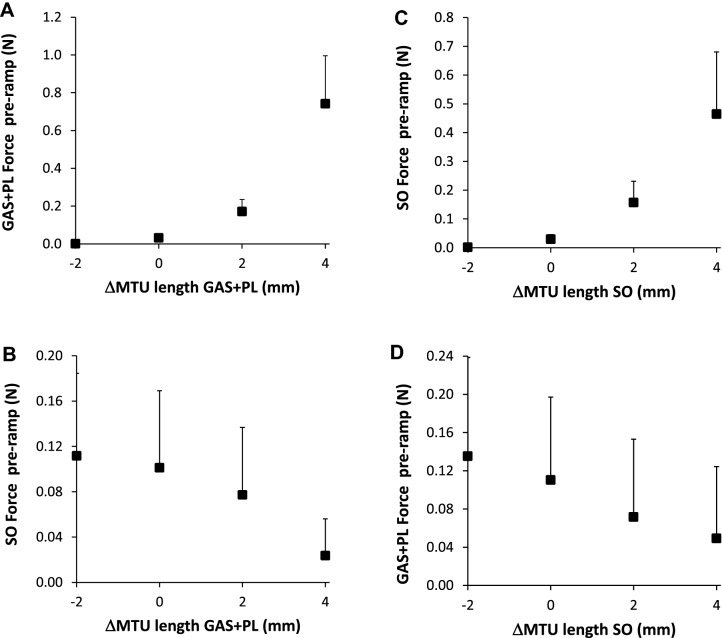
Distal forces of GAS + PL and SO before the onset of the ramp-hold-release protocol. **A** Distal GAS + PL and **B** SO forces plotted as a function of GAS + PL MTU length. The MTU length of SO was kept constant at Lref. (C) Distal SO and (D) GAS + PL forces plotted as a function of SO MTU length. The MTU length of GAS + PL was kept constant at Lref. ΔMTU length 0 corresponds to reference length (Lref). Values are shown as mean + SD (*n* = 8 for effects of lengthening GAS + PL; *n* = 8 for effects of lengthening SO)

Lengthening GAS + PL by 6 mm (from Lref – 2 mm to Lref + 4 mm) resulted not only in an increase in the force exerted at their own distal tendons prior to the RHR protocol (Fig. 3A, *p* < 0.001), but also in a decrease in the force exerted at the distal tendon of SO (Fig. 3B, p < 0.001). Also when lengthening SO, an increase in the force exerted at SO tendon (Fig. 3C,  *p* < 0.001) was accompanied by a decrease in the force exerted at LG + PL tendons (Fig. 3D,  *p* = 0.001). Similar effects of changes in MTU length were found for the peak force exerted at the end of the stretch and during the hold-phase of the RHR protocol (data not shown). These results confirmed that the imposed experimental conditions involved changes in force transmitted via epimuscular pathways between GAS + PL and SO muscles, as described previously (Maas 2009).

#### Effects of synergist lengthening on firing behavior of LG and SO tendon organs

**Fig. 4:**
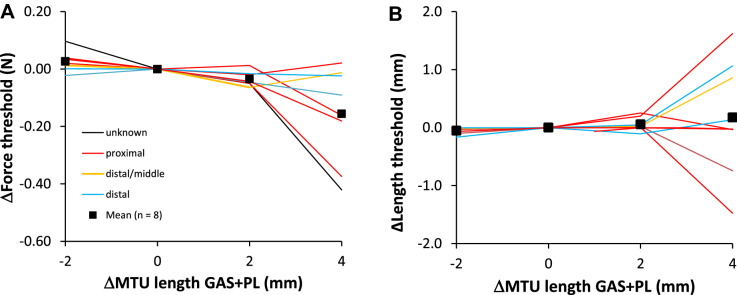
Effects of GAS + PL lengthening on force (**A**) and length (**B**) threshold of SO tendon organs. Each parameter is plotted as a function of GAS + PL MTU length, expressed as the deviation from Lref (ΔMTU length). To compare individual cells, the value at Lref was subtracted from the value at all other lengths to obtain a delta (Δ) value. Mean values (*n* = 8) are shown as black squares. Different colors indicate the proximal–distal position of each tendon organ within the muscle belly (legend in **A**)

SOLEUS. Lengthening GAS + PL distally from Lref – 2 mm to Lref + 4 mm resulted in a significant (p = 0.035) decrease in the force threshold (Fig. 4), by 0.18 N; a reduction of 50% relative to the value at Lref (Table 1). Note that the change in force threshold appeared to be more substantial in the proximally located tendon organs (red lines in Fig. 4A). Post hoc analysis revealed that the force threshold differed between ΔMTU − 2 mm and + 2 mm (*p* = 0.035), as well as between 0 and + 2 mm (*p* = 0.033). The length threshold was not affected by GAS + PL length (p = 0.599).

**Fig. 5:**
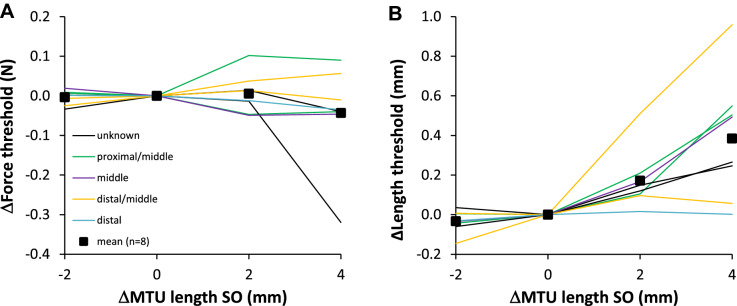
Effects of SO lengthening on force (**A**) and length (**B**) threshold of LG tendon organs. Each parameter is plotted as a function of SO MTU length, expressed as the deviation from Lref (ΔMTU length). To compare individual cells, the value at Lref was subtracted from the value at all other lengths to obtain a delta (Δ) value. Mean values (n = 8) are shown as black squares. Different colors indicate the proximal-distal position of each tendon organ within the muscle belly (legend in A)

*Lateral Gastrocnemius* The force threshold of LG tendon organs was not affected by SO length (*p* = 0.371). However, lengthening SO from Lref − 2 mm to Lref + 4 mm resulted in a significant (*p* = 0.005) increase in the length threshold (Fig. 5), by 0.42 mm (i.e. 35% higher than the value at Lref, see Table 1). Post hoc analysis revealed that the length threshold differed between ΔMTU − 2 mm and + 2 mm (p = 0.024), as well as between − 2 mm and + 4 mm (*p* = 0.027).

### Experiment 2: Effects of relative position of LG + PL

#### Characteristics of recorded tendon organ afferents

Axonal conductional delays and ramp parameters, measured with all muscles at reference length and position (i.e., ankle and knee angle at 90°; Table 2), were consistent with those described previously (see above). The approximate location within the muscle belly could be identified for all except 2 out of 11 LG afferents, but all afferents were included in the analysis.

**Table 2:** Firing of tendon organs in lateral gastrocnemius muscle (n = 11) and tendon force parameters measured before and during proximal and distal ramp-hold-releases of LG + PL at reference length (Lref) and position (Pref)

Proximal–distal position	2d, 2d/m, 3 m, 0 pm, 2p, 2nc	
Axonal conductional delays (ms)	1.9 ± 0.2	
Parameters at Pref	Distal ramps	Proximal ramps
Force threshold, proximal (N)	0.56 ± 0.47	0.63 ± 0.49
Length threshold (mm)	0.86 ± 0.68	0.94 ± 0.69
Peak frequency (pps)	77 ± 44	95 ± 114
Static index (pps)	22 ± 10	20 ± 11
Pre-ramp force (N)	0.23 ± 0.12	0.44 ± 0.61
Max ramp force (N)	2.74 ± 1.85	2.26 ± 1.63

*n* number of afferents, *nc* not classified, positions: *d* distal, *m* middle, *p* proximal

Values are means ± SD

#### Effects of muscle relative position on proximal LG + PL and distal LG + PL + SO forces

**Fig. 6:**
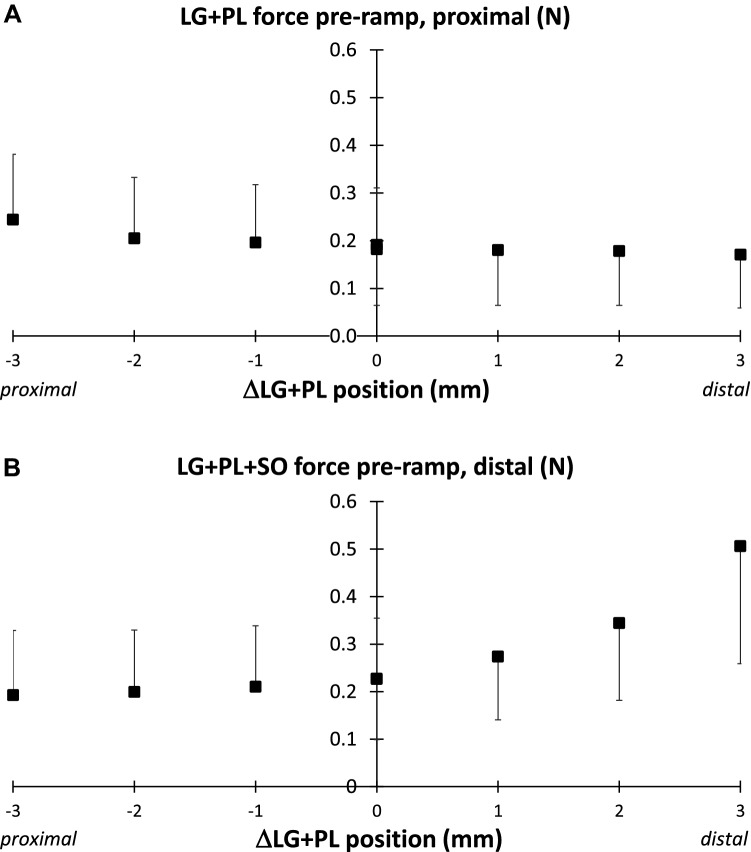
Proximal forces of LG + PL and distal forces of LG + PL + SO before the onset of the ramp-hold-release protocol. **A** Proximal LG + PL and **B** LG + PL + SO forces plotted as a function of LG + PL muscle relative position. The MTU length of LG + PL was kept constant at Lref. ΔLG + PL position 0 corresponds to reference length (Lref) and position (Pref). Note that displacements of the distal tendons caused changes in MTU length of SO. Values are shown as mean + or − SD (*n* = 11)

Displacing LG + PL from the most proximal position (-3 mm) to the reference position (0 mm) and to the most distal position (+ 3 mm) decreased force at the proximal LG + PL tendon (from 0.24 ± 0.14 to 0.17 ± 0.11 N) exerted prior to the RHR protocol (Fig. 6A; *p* < 0.001 for proximal positions and p = 0.010 for distal positions), despite the fact that LG + PL muscle–tendon unit length was kept constant. In contrast, forces exerted at the distal tendons of LG + PL + SO increased (from 0.19 ± 0.14 to 0.51 ± 0.25 N) with LG + PL moving in distal direction (Fig. 6B; *p* < 0.001 for both proximal and distal positions). Similar effects of changes in LG + PL relative position were found for the peak force exerted at the end of the stretch and during the hold-phase of the RHR protocol (data not shown). The force changes exerted at the proximal tendons of LG + PL, which were kept at a constant length, confirmed that the imposed experimental conditions involved changes in force transmitted via epimuscular pathways. Because the displacements of the distal tendons involved changing the MTU length of SO, the changes in the force exerted at the distal tendons of LG + PL + SO should at least partly be attributed to the effects of SO length.

#### Effects of LG + PL muscle relative position on firing behavior of LG tendon organs

**Fig. 7:**
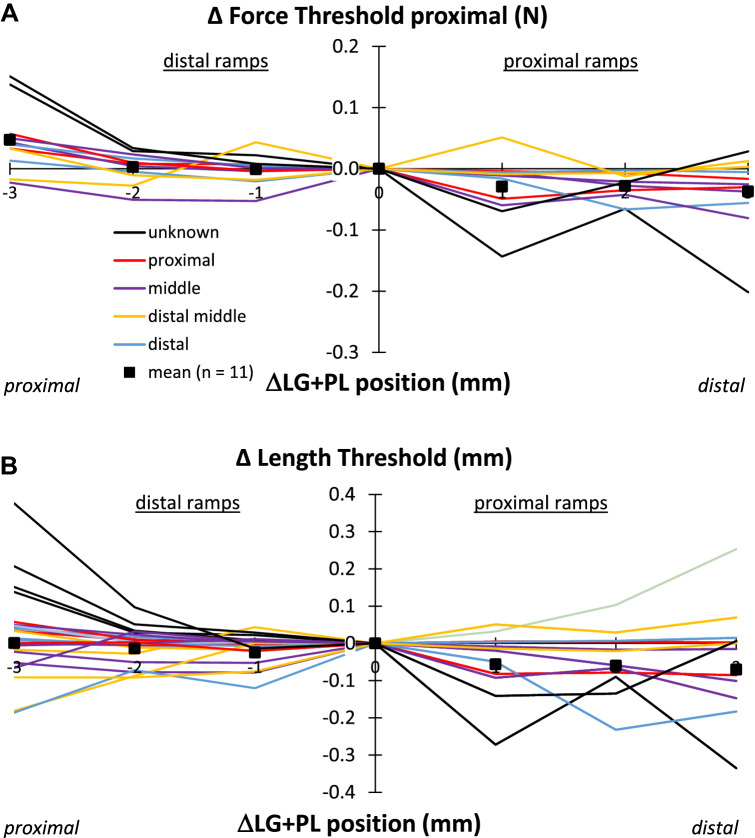
Effects of LG + PL muscle relative position on force (**A**) and length (**B**) threshold of LG tendon organs. Each parameter is plotted as a function of LG + PL muscle relative position, expressed as the deviation from the reference position (ΔLG + PL position). To compare individual cells, the value at Lref was subtracted from the value at all other lengths to obtain a delta (Δ) value. Mean values (*n* = 11) are shown as black squares. Different colors indicate the proximal–distal position of each tendon organ within the muscle belly (legend in A)

Displacing LG + PL from the most proximal position (− 3 mm) to the reference position (0 mm) resulted in a significant (*p* = 0.007) decrease in the force threshold (Fig. 7A), by 0.05 N (i.e. 9% of value at Pref, see Table 2). Post hoc analysis revealed that the force threshold differed between Δ*P* = − 3 mm and Δ*P* = − 2 mm. The displacement of LG + PL from the reference position (0 mm) to the most distal position (+ 3 mm) decreased the force threshold by another 0.04 N (i.e. 6%), but this was not significant (p = 0.095). Note that between position 0 mm and + 3 mm also the proximal LG + PL force did change less than between − 3 and 0 mm (Fig. 6A). For the length threshold, no significant effects of LG + PL position was found (*p* = 0.645 and p = 0.061 for the proximal and distal positions, respectively).

## Discussion

Studies on epimuscular myofascial force transmission have focused mainly on its mechanical consequences. Previously, we showed the effects of connective tissue linkages between two neighboring muscles on the firing behavior of muscle spindles (Smilde et al. 2016). These results indicated that muscle spindles may not only signal length changes of the muscle in which they are located but also local length changes that occur as a result of changing the length and hence, relative position of a synergistic muscle. In the present study, we investigated the effects of such intermuscular linkages on feedback from tendon organs. Our results were in agreement with our hypothesis that tendon organ firing is affected by changes in length and/or relative position of adjacent synergistic muscles. This suggests that tendon organs can provide the central nervous system with information regarding epimuscular myofascial forces.

In this study, we manipulated the length or position of muscle(s) adjacent to the muscle in which the tendon organ was located. As reported previously (Maas 2019), lengthening an adjacent muscle distally will attract force to the lengthened muscle and, thereby, will result in a decrease in force at the distal tendon of the restrained muscle (Fig. 3). We found a decrease in force threshold for the majority of SO tendon organs, but not for tendon organs in LG. Thus, SO tendon organs started firing at a lower force exerted on its distal tendon. This suggests that the tendon organs were preloaded by the intermuscular linkages applying a distally directed load on SO muscle (Fig. 1A). As a consequence, a smaller change in force was required to bring the tendon organs beyond their threshold for firing. That this was not observed for most LG tendon organs may be explained by the location of the sample of tendon organs. In SO this was biased towards proximal, while in LG most tested tendon organs were located distally. Distally located tendon organs could actually be unloaded by the distally directed epimuscular loads. This is in agreement with the increase in the length threshold (Fig. 5B), indicating that more lengthening of LG was required for eliciting firing of its tendon organs. Another difference between SO and LG muscles is their architecture. The same myofascial loads may affect the pennate LG differently than the parallel fibered SO. It is also possible that the LG tendon organs sampled were located at a greater distance from the interface between these muscles than those of SO. Because the number of tendon organs sampled from each location is too small, and it is difficult to accurately assess tendon organ location, we must be cautious drawing conclusions about local effects. We can conclude, however, that tendon organs can sense epimuscular loads.

For each experiment, a relatively small sample of tendon organs (*n* = 8–11) was tested. Our measurements did not only involve the classification of afferent type and the assessment of the classical firing characteristics (see Tables 1 and 2), but also of the effects of muscle length and relative position. This requires stable recordings for up to 20 min and, hence, less tendon organs could be tested per animal. Despite this, we observed statistically significant effects for our main outcome parameters, as most tendon organs responded to the manipulation in a similar way. Even though myofascial forces are present in passive muscle conditions (Maas 2019), their amplitude is substantially higher during active muscle conditions (Tijs et al. 2016). This means that manipulating muscle relative position will cause greater changes in force and, hence, also greater changes in tendon organ firing if the muscles in the present study would have been in active state. Also the firing of tendon organs is lower during passively stretching a muscle compared to during an active contraction (Vincent et al. 2017). Note however that, in contrast to some observations in cats (Jami 1992), the firing of tendon organs in response to physiologically relevant stretches of passive muscle appears to be robust in rats (Vincent et al. 2017). A comparison of tendon organ firing characteristics at a reference position in Experiment 1 (see Table 1) with those of muscle spindles obtained during similar conditions previously (Smilde et al. 2016) revealed that both force (1.4 and 3.3 fold for SO and LG, respectively) and length thresholds (2.2 and 3.0 fold for SO and LG, respectively) are higher, and the peak firing frequency is lower (0.6 and 0.7 fold for SO and LG, respectively) for tendon organs. The muscle conditions studied in Experiment 1 were different than those found during normal movements. Stretches were imposed on GAS + PL and SO muscles individually while during normal movements of the ankle joint, the length of these muscles will be affected to a similar extent. That is why we added Experiment 2 in which physiological changes in muscle length and relative position were imposed, simulating ankle joint rotations (i.e. lengthening LG + PL and SO simultaneously) or knee joint rotations (i.e. lengthening LG + PL exclusively). The results were qualitatively similar between Experiment 1 and 2.

Tendon organs are located at both the proximal and distal fiber ends along the whole length of the aponeuroses (Swett and Eldred 1960; Zelena and Soukup 1983). Also muscle spindles are not evenly distributed within the muscle belly (Arendt and Asmussen 1974; Swett and Eldred 1960). Why skeletal muscles have so many spatially distributed sensors is still an enigma (Nordin et al. 2017; Windhorst 2008). According to the ensemble encoding theory, ensembles of muscle sensors encode information about the muscle state more precisely than individual sensors (Bergenheim et al. 1996; Loeb and Marks 1985; Mileusnic and Loeb 2009; Stein 1967). Alternatively, muscle sensors provide specialized feedback that depends on their location within the muscle. There are many conditions that involve non-uniform strains and stresses within muscles. Hence, differential feedback is expected, for example, during asynchronous excitation of muscle compartments (Weeks and English 1985; Windhorst et al. 1989) or due to forces transmitted via connective tissue linkages between adjacent muscles (Huijing 2007; Maas 2009; Maas and Sandercock 2010). It has been shown that many muscle spindles respond selectively to contractions of single muscle compartments, called sensory partitioning (Windhorst et al. 1989). Also, stronger connections have been found between muscle spindles and motoneurons of the same compartment than those between muscle spindles and motoneurons linked to different compartments, named reflex partitioning (Windhorst et al. 1989). Some muscle spindles were found to respond to length changes of neighboring muscles (Smilde et al. 2016). And in the present study, we show that myofascial loads affect some but not all tendon organs. Thus, suggesting that tendon organs and, hence, the central nervous system may sense local stresses varying within the muscle belly. These local sensorimotor circuits may serve to limit non-uniformities to prevent excessive strains and stresses and, hence, muscle injury. It has also been hypothesized that local information about muscle fiber stress may be used for joint-specific force feedback from muscles crossing two joints (Maas 2009).

We conclude that tendon organs can provide the central nervous system with information about local stresses caused by epimuscular myofascial forces. Further studies should investigate if and how such anatomical organization is used for the neural control of movement.

## Data Availability

The data that support the findings of this study are available from the corresponding author upon reasonable request.
